# Safe Follow-Up after Endovascular Aortic Repair with Unenhanced MRI: The SAFEVAR Study

**DOI:** 10.3390/diagnostics13010020

**Published:** 2022-12-21

**Authors:** Francesco Secchi, Davide Capra, Caterina Beatrice Monti, Nazanin Mobini, Maria Del Mar Galimberti Ortiz, Santi Trimarchi, Daniela Mazzaccaro, Paolo Righini, Giovanni Nano, Francesco Sardanelli

**Affiliations:** 1 Department of Biomedical Sciences for Health, Università degli Studi di Milano, 20133 Milan, Italy; 2 Unit of Radiology, IRCCS Policlinico San Donato, San Donato Milanese, 20097 Milan, Italy; 3 Postgraduation School in Radiodiagnostics, Università degli Studi di Milano, 20122 Milan, Italy; 4 Department of Vascular Surgery, Fondazione IRCCS Cà Granda Ospedale Maggiore Policlinico, 20122 Milan, Italy; 5 Clinical and Community Sciences Department, Università degli Studi di Milano, 20122 Milan, Italy; 6 Unit of Vascular Surgery, IRCCS Policlinico San Donato, San Donato Milanese, 20097 Milan, Italy

**Keywords:** computed tomography angiography, magnetic resonance imaging, endovascular procedures, endoleak, aortic aneurysm

## Abstract

We aimed to investigate whether unenhanced magnetic resonance imaging (MRI) could represent a safe and highly sensitive tool for endoleak screening in patients treated with endovascular aneurysm repair (EVAR) using computed tomography angiography (CTA) as a reference standard. Patients who underwent CTA for EVAR follow-up at our institution were prospectively enrolled. All MRI examinations were performed with a 1.5 T unit. The true-FISP and HASTE sequences of the MRI scans were assessed for the presence of hyperintensity within the aneurysm sac outside the graft, whereas phase-contrast through-plane sequences were used for blood flow quantification. We included 45 patients, 5 (11%) of whom were female. The median age was 73 years (IQR 68–78 years). Among our patients, 19 (42%) were positive for endoleaks at CTA, of whom 13 (68%) had type II endoleaks and 6 (32%) had type I endoleaks. There were no significant differences in age, sex, aneurysm type, prosthesis type, or contrast-to-noise ratio between hyperintensity and thrombus between patients with and without endoleaks (*p* > 0.300). The combined evaluation of true-FISP and HASTE yielded 100% sensitivity (95% CI: 79–100%) and 19% specificity (95% CI: 7–40%). Patients with a positive CTA had a median thrombus flow of 0.06 L/min (IQR 0.03–0.23 L/min), significantly greater than that of patients with a negative CTA (*p* = 0.007). Setting a threshold at 0.01 L/min, our MRI protocol yielded 100% sensitivity, 56% specificity, and an AUC of 0.76 (95% CI 0.60–0.91). In conclusion, unenhanced MRI has perfect sensitivity for endoleak detection, although with subpar specificity that could be improved with phase-contrast flow analysis.

## 1. Introduction

The surgical management of aortic aneurysms has changed radically over the last three decades since the introduction of endovascular aneurysm repair (EVAR) in the early 1990s [1]. Compared to open repair surgery, EVAR is less invasive and displays lower periprocedural morbidity and mortality [2]. However, two recent meta-analyses by Li et al. [3] and Antoniou et al. [4] hint at the fact that this early advantage may be partially lost over time, with EVAR patients experiencing higher rates of reintervention and higher aneurysm-related mortality after 8 years [4]. In fact, EVAR carries an inherent risk of postprocedural complications, namely, graft migration or rupture, obstruction, infections, and endoleaks, the latter being the most common [5].

An endoleak is defined as the persistence of blood flow within the aneurysm sac after EVAR [6], and it can be classified into five main categories [7]: type I, due to leakage from the proximal or distal insertion point of the graft; type II, sustained by retrograde blood flow from aortic branches into the aneurysmatic sac; type III, caused by graft wall breaches such as tears; type IV, due to graft wall porosity; and type V, represented by aneurysmatic sac enlargements without any evidence of blood leakage. Endoleaks are estimated to occur in 15% to 25% of patients during the first month after the procedure [5] but can also present months or years after EVAR [8]. As a consequence, lifelong surveillance is recommended. 

While all current guidelines recommend baseline imaging via computed tomography angiography (CTA) with the intravenous administration of an iodinated contrast agent within a month of EVAR (class I recommendation, level B evidence) [6,9], there is no widespread consensus concerning the timing of periodic CTA surveillance, ranging from 1-year [6] to 5-year intervals [9] when no endoleaks are detected. Triphasic CTA, including unenhanced, arterial, and delayed acquisitions, is currently regarded as the standard of care for the detection of endoleaks. However, this practice involves a non-negligible cumulative radiation dose [10,11,12,13]. In addition, the risk of contrast-induced nephropathy has to be carefully taken into account, considering that up to 36% of EVAR patients develop renal impairment within three years of the procedure [14]. 

Hence, contrast-enhanced magnetic resonance imaging (MRI) angiography has been proposed as a radiation-free alternative to CTA, showing a high sensitivity in endoleak detection, especially for type II endoleaks [5,11,15,16]. However, this technique employs gadolinium-chelate, which has shown the potential for retention in human tissues, particularly in the brain, raising concerns in the medical community over the last few years [17,18,19].

Interestingly, MRI enables the evaluation of the aorta of patients who underwent EVAR without administering a contrast agent using a variety of unenhanced sequences, further reducing the impact of imaging follow-up in these frail patients, avoiding both radiation exposure and contrast agent administration. In particular, the true fast imaging with steady-state precession (true-FISP) sequence is a fast gradient-echo sequence that allows free-breathing acquisitions and good visualization of abdominal vessels [20], with potentially high sensitivity in the detection of endoleaks [21]. Similarly, half-Fourier acquisition single-shot turbo spin-echo (HASTE) is a black-blood T2-weighted sequence that requires short acquisition times, typically under one minute, offering good visualization of the aortic anatomy and walls [22]. Finally, a phase-contrast sequence allows the analysis of the flow in the excluded aneurysmatic sac [23]. 

Thus, we aimed to investigate whether unenhanced MRI can be adopted as a tool for screening for endoleaks in patients treated with EVAR, assessing the diagnostic performance of morphological and flow sequences for the detection of endoleaks, using CTA as a standard of reference. 

## 2. Materials and Methods

### 2.1. Study Population

This study was approved by the local Ethics Committee (Ethics Committee of San Raffaele Clinical Research Hospital; protocol code “SAFEVAR”, approved on 4 February 2016). Each patient provided written informed consent. This study was partially supported by Ricerca Corrente funding from the Italian Ministry of Health to IRCCS Policlinico San Donato. 

Patients who underwent CTA or MRI for EVAR follow-up at our institution were prospectively enrolled. Exclusion criteria were contraindications to MRI. Patients unable to complete the MRI examination or with extensive artifacts on MRI were excluded ex post. 

### 2.2. Image Acquisition

The CTA scanning protocol was performed on a 64-detector row scanner (Somatom Definition, Siemens Healthineers, Erlangen, Germany) with a voltage of 120 kV, a tube current ranging from 275 to 300 mAs depending on an automatic exposure control system (CARE Dose 4D, Siemens Healthineers, Erlangen, Germany), 0.5 s rotation speed, and pitch 0.65B30f medium smooth kernel using an abdominal window, with a slice thickness of 0.75 mm. Moreover, iopamidol (Iopamiro 370; 370 mg I/mL; Bracco Imaging, Milan, Italy) was administered based on the patient’s total body weight (1 mL/kg). This contrast agent was injected intravenously through a 20-gauge needle using an automatic power injector (EmpowerCTA Contrast Injection System, Bracco Imaging, Milan, Italy) at a rate of 4 or 5 mL/s, followed by 50 mL of saline solution at the same rate.

All MRI examinations were performed with a 1.5 T unit (Magnetom Aera, Siemens Healthineers, Erlangen, Germany) and an 18-channel phased-array surface coil. According to the aortic aneurysm location, the thorax or the abdomen was studied using three electrocardiographically gated sequences, described in Table 1. Phase-contrast through-plane sequences were used for blood flow quantification on images perpendicular to the vessel of interest, with a velocity encoding from 150 cm/s to 350 cm/s. In the presence of aliasing, we increased the velocity encoding, adding 50 cm/s for each new sequence step by step until the complete disappearance of the aliasing artifact.

### 2.3. Image Analysis

All CTA datasets were reviewed by a radiologist with 12 years of experience in cardiovascular imaging (R1) for the detection of endoleaks. 

True-FISP and HASTE sequences of the MRI scans were assessed for the presence of hyperintensity within the aneurysm sac outside the graft by both R1 and a medical student (R2) with one year of experience in cardiovascular imaging to assess reproducibility. If any hyperintensity was found, the reader placed two regions of interest (ROIs) in the thrombus and hyperintensity on the slice where the hyperintensity itself was most evident. If no hyperintensity was found, only one ROI was placed in the thrombus. For each ROI, the signal intensity and its standard deviation were registered. Flows in both the thrombus (if present) and in the endograft lumen were measured on the phase-contrast sequences.

### 2.4. Statistical Analysis

The sensitivity, specificity, positive predictive value (PPV), and negative predictive value (NPV) of MRI were calculated using CTA as a reference standard. Each MRI scan was considered positive if either the true-FISP or HASTE sequence showed hyperintensity near the EVAR site. The diagnostic performance of MRI was calculated separately for morphological sequences and flow sequences. Furthermore, agreement between the two sequences and the two readers was appraised using Cohen’s κ and interpreted according to Landis and Koch [24]. 

Data are reported as the median and interquartile range (IQR) due to the small sample size. Potential differences between patients with and without endoleaks in terms of age, type of aneurism, type of prosthesis, contrast-to-noise ratio (CNR) between hyperintensity and the lumen, and thrombus flow were appraised. Such differences were analyzed using the Mann–Whitney *U* test for continuous variables and with Fisher χ^2^ for categorical variables. If significant differences were found, receiving operator characteristic (ROC) analyses were performed to find the threshold that better maximized the diagnostic potential of the studied variable.

Statistical analyses were performed with R 3.5.2 (R Foundation for Statistical Computing, Vienna, Austria), and *p*-values ≤ 0.05 were considered indicative of statistical significance [25].

## 3. Results

### 3.1. Study Population

A total of 49 patients underwent CTA for post-EVAR follow-up during the study timeframe (between February 2015 and December 2018) and were enrolled in our study. Subsequently, one patient who could not complete the MRI examination and three who presented extensive artifacts on MRI were excluded. Thus, our study sample consisted of 45 patients, 5 (11%) of whom were females. The median age was 73 years (IQR 68–78 years); patients’ characteristics are reported in Table 2.

Among the 45 included patients, 19 (42%) were positive for endoleaks at CTA, with 13 (68%) of them presenting with type II endoleaks and the remaining 6 (32%) with type I endoleaks. There were no significant differences in age, sex, type of aneurysm, type of prosthesis, or CNR between hyperintensity and thrombus between patients with and without endoleaks (*p* ≥ 0.300).

### 3.2. Morphological MRI Diagnostic Performance 

**Figure 1:**
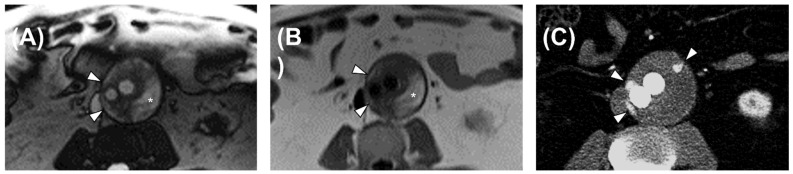
An example of a true-positive case, a 68-year-old male patient. (**A**) True-FISP sequence: the white arrowheads indicate hyperintensities related to the endoleak, whereas * denotes the hyperintensity of the blood clot in the aneurysmatic sac. (**B**) HASTE sequence: the white arrowheads indicate hyperintensities related to the endoleak, whereas * denotes the hyperintensity of the blood clot in the aneurysmatic sac. (**C**) CTA: arterial phase acquisition. The arrowheads indicate three hyperdense areas suggesting endoleak, one of which is not clearly visible in the RM images.

**Figure 2:**
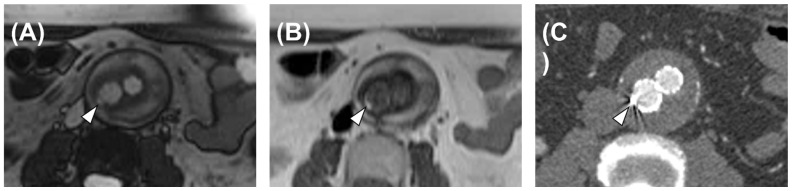
An example of a false-positive case, a 78-year-old male patient. (**A**) True-FISP sequence: hyperintensity can be observed next to the wall of the endograft (arrowhead). (**B**) HASTE sequence: hyperintensity can be observed next to the wall of the endograft (arrowhead). (**C**) CTA: arterial phase acquisition. A metallic artifact can be observed in correspondence with RM hyperintensities (arrowhead), possibly causing the misinterpretation of the RM images.

True-FISP scans were deemed suspicious for endoleaks in 40 cases, thus yielding 100% sensitivity (95% CI: 79–100%), 19% specificity (95% CI: 7–40%), 48% PPV (95% CI: 32–64%), and 100% NPV (95% CI: 46–100%). The contingency table for the calculation of diagnostic performance is reported in Table 3. An example of a true-positive case is reported in Figure 1, whereas a false-positive case is depicted in Figure 2.

HASTE sequences were positive in 38 cases, showing 100% sensitivity (95% CI: 79–100%), 27% specificity (95% CI: 12–48%), 50% PPV (95% CI: 34–66%), and 100% NPV (95% CI: 56–100%), displaying marginal improvement compared to true-FISP assessment. The contingency table is reported in Table 3**.**

Thus, the combined evaluation of true-FISP and HASTE yielded 100% sensitivity (95% CI: 79–100%), 19% specificity (95% CI: 7–40%), 48% PPV (95% CI: 32–64%), and 100% NPV (95% CI: 46–100%). The contingency table is reported in Table 3. The two readers showed moderate agreement on the overall MRI assessment of endoleaks, with a Cohen’s κ of 0.500 (*p* < 0.001). The HASTE sequence showed the highest concordance among the two readers, yielding a Cohen’s κ of 0.697 (*p* < 0.001), whereas true-FISP displayed a κ of 0.500 (*p* < 0.001). For both sequences, all discordant cases among readers were negative for endoleaks at CTA. 

### 3.3. Flow MRI Diagnostic Performance 

Patients with a positive CTA had a median thrombus flow of 0.06 L/min (IQR 0.03–0.23 L/min), significantly greater than that of patients with a negative CTA (0.01 L/min (IQR 0.03–0.04 L/min), *p* = 0.007). A cutoff of 0.03 L/min yielded an area under the curve (AUC) of 0.81 (95% CI 0.60–0.96), with a sensitivity of 82% and a specificity of 75% for detecting endoleaks. To preserve the 100% sensitivity, the cutoff should be set at 0.01 L/min, obtaining a specificity of 56% and an AUC of 0.76 (95% CI 0.60–0.91). A comparison of further MRI features between patients with a positive and negative CTA is reported in Table 4.

## 4. Discussion

This study assessed the diagnostic performance of three unenhanced, i.e., “non-contrast”, MRI sequences for the detection of endoleaks after EVAR, obtaining 100% sensitivity and NPV, although with subpar specificity and PPV. 

Currently, post-EVAR follow-up is mainly based on CTA, as per American and European guidelines [6,9]. However, repeated exposure to CTA, especially considering triphasic studies, raises concerns about radiation safety. Brambilla et al. [26] reported an annual mean cumulative exposure of 129 ± 77 mSv in a group of 71 patients undergoing post-EVAR follow-up, which is over the 100 mSv threshold. This finding would imply a necessity for a thorough evaluation of the risk/benefit balance [27], although the study data are from a historical database, including examinations performed using older scanners, which would lead to higher radiation doses than current ones. Nevertheless, novel approaches should aim for dose reduction, as recently suggested by the European Society of Radiology EuroSafe Imaging [27]. In this light, an initial screening for endoleaks without radiation exposure through MRI would be advisable. 

The relevant issue posed by our results is the imbalance between high sensitivity and low specificity, which has significant implications for translating them into clinical practice due to a high rate of false-positive patients. Indeed, regarding the sensitivity and NPV of unenhanced MRI sequences, a preliminary study by Resta et al. [21] showed 100% sensitivity and optimal inter-reader reproducibility for endoleak detection with true-FISP MRI. In fact, thanks to its hybrid T1/T2 weighting, true-FISP offers high contrast between different anatomical structures, showing hyperintense signals inside vessel lumens, independently of the blood flow velocity or direction. Conversely, HASTE MRI, used in addition to true-FISP, provides black-blood images of the volume of interest, thus improving the visualization of aortic walls, with an acquisition time of a few seconds [28]. Indeed, our study observed 100% sensitivity for both true-FISP and HASTE sequences, with the latter correctly classifying two more negative patients than the former, reaching slightly better specificity. Of note, our unenhanced protocol can be performed within a short examination time, usually under 10 min. Other MRI studies showed high sensitivities for endoleaks using contrast-enhanced sequences [16,29,30]. However, non-contrast MRI protocols would have the advantages of being faster and more economical and, furthermore, would spare patients the administration of gadolinium-chelates, which have the potential for retention in human tissues [17,18,19].

The low specificity displayed by both sequences (19% for true-FISP and 27% for HASTE) may be due to patterns of signal hyperintensities that could be observed in the thrombus through various stages of clot formation, mimicking an endoleak [31]. Thus, we might expect MRI specificity to increase over time, as the organization of tissue within the excluded aneurysmatic sac can take over six months [31]. Such a low specificity may hinder the advantages of fast MRI screening for endoleaks, returning a high rate of false-positive patients who would have to undergo additional examinations. Nevertheless, such patients would already undergo CTA according to current recommendations [6,9]. Hence, even in the presence of a high number of false positives, introducing MRI screening for endoleaks would allow the sparing of non-negligible radiation doses for patients with a negative MRI assessment for endoleaks. 

To mitigate the number of false-positive cases, we also investigated the blood flow in the thrombus with a phase-contrast sequence, and we observed an average flow of 0.06 L/min in patients with a positive CTA. We therefore set a threshold of 0.01 L/min, yielding 100% sensitivity and 56% specificity with our MRI protocol. It would thus be possible to improve the PPV of MRI without the need for contrast administration, perhaps keeping short acquisition times by using parallel acquisition [23]. Moreover, a recent study from Kawada et al. [32] suggests the possibility of evaluating various additional qualitative features of the aneurysmal sac to improve the specificity of non-contrast MRI angiography for the detection of endoleaks. 

Thus, our approach suggests the possibility of a new strategy for post-EVAR follow-up of the first screening for endoleaks using non-contrast MRI, effectively ruling out endoleaks in the case of a negative assessment. If there is a suspected endoleak on non-contrast MRI, patients could then undergo CTA.

While our study focused on unenhanced MRI, a further scenario could be offered by contrast-enhanced MRI angiography. A systematic review published in 2013 [15] suggested that MRI angiography could be more sensitive than CTA in endoleak detection, unveiling 132 additional endoleaks in 369 patients compared to CTA. However, it was partially based on studies that used intravenous blood pool contrast agents, which have since been discontinued [33,34,35], and hence, its conclusions might overestimate the sensitivity of MRI angiography in everyday clinical practice. 

Another radiation-free alternative to CTA for safe post-EVAR surveillance could be represented by color duplex ultrasonography (DUS). Indeed, DUS is considered an accurate modality to detect sac enlargement and endoleaks (particularly types I and III) and has been suggested for annual post-EVAR surveillance, albeit only in low-risk patients [6,9]. However, DUS is dependent on operator experience and on patients’ characteristics, it does not allow a reliable assessment of graft position variations, and, most notably, sac diameter measurements are not directly comparable to CT measurements [9]. Therefore, we hypothesize that MRI surveillance could be beneficial particularly in intermediate-risk patients who have known type II endoleaks and who need to the monitor sac diameter for expansion or shrinkage. In those patients, the possibility of a direct comparison between CT and MRI measurements may be an advantage.

The most obvious concern about an approach based on MRI would be the potential safety issues posed by aortic endografts. However, aortic stent grafts are generally considered MRI-safe [36]. In our study, no graft posed a contraindication to MRI, and only three patients with stainless steel grafts presented with extensive artifacts that undermined the readability of the exam. Therefore, we would recommend post-EVAR MRI screening only in patients with nitinol stents, as already suggested by Alerci et al. [30]. Such a strategy could allow a substantial reduction in the radiation dose delivered to those patients and potentially avoid contrast administration in a population of patients that is already at increased risk for renal dysfunction [14]. 

This study presents some limitations. First, we did not perform a comparison between DUS and MRI. However, our main purpose was to evaluate the diagnostic performance of non-contrast MRI for endoleak detection against CTA, which is currently considered the main reference standard for EVAR follow-up. Second, the evaluation of MRI images was performed by two readers with different degrees of experience. Nevertheless, the lowest inter-reader agreement was κ = 0.500 for the true-FISP sequence, suggesting an overall acceptable concordance of MRI post-EVAR imaging, even among readers with substantial differences in experience. Furthermore, the higher agreement reached by HASTE seems to indicate that black-blood imaging might be easier to interpret by readers with less experience, perhaps due to the better differentiation of extraluminal hyperintensities from the blood pool. Third, our study was based on outpatients, and therefore, an analysis at standardized timepoints was not possible. Moreover, a further follow-up of the studied cohort was not available, thus not allowing the evaluation of the potential clinical impact of the MRI endoleak assessment on patients’ management. Fourth, our population included only 45 patients with 19 cases of endoleaks, most of which were type II endoleaks, and the remaining were type I endoleaks. Thus, our findings may not be generalizable to other types of endoleaks. Further studies on larger populations are warranted to address these last two critical aspects.

## 5. Conclusions

In summary, this study confirms the high sensitivity of MRI for endoleak detection after EVAR, even with fast, non-contrast sequences. These findings advocate for the use of HASTE and true-FISP MRI, possibly along with phase-contrast acquisitions for flow detection, as a first-line screening tool for endoleaks, reverting to further contrast-enhanced MRI techniques or CTA only if MRI cannot rule out an endoleak. This strategy would allow a reduction in both radiation and contrast doses in a population of patients that is destined for life-long radiological surveillance.

## Figures and Tables

**Table 1:** MRI acquisition parameters.

Acquisition Parameters	True-FISP	HASTE	Phase-Contrast
Time of repetition (ms)	237	590	37.12
Time of echo (ms)	1.15	44	2.47
Section thickness (mm)	7	6	6
Flip angle (°)	80	145	20
Field of view (mm)	400	400	340
Acquisition time (s)	120	22	15

**Table 2:** Cohort characteristics.

Parameter	Value
Female sex (n, %)	5 (11)
Male sex (n, %)	40 (89)
Total	45 (100)
Age (median, IQR)	73 (68–78)
Interval EVAR-CTA (days; median, IQR)	96 (61–562)
Interval EVAR-MRI (days; median, IQR)	92 (57–372)
Interval CTA-MRI (days; median, IQR)	0 (0–0)
Type of lesion	
AAA (n, %)	27 (60)
TAA (n, %)	6 (13)
T-AAA (n, %)	7 (16)
Iliac AA (n, %)	3 (7)
Type B dissection (n, %)	2 (4)
Total	45 (100)
Lumen diameter (mean, SD)	56.9 (13.5)
Thrombus diameter (mean, SD)	16.1 (8.6)
Stent material	
Nitinol (n, %)	36 (80)
Unknown (n, %)	9 (20)
Total	45 (100)
Type of endoleak	
Type I (n, %)	6 (32)
Type II (n, %)	13 (68)
Total (n, %)	19 (100)

AAA, abdominal aortic aneurysm; CTA, computed tomography angiography; EVAR, endovascular aneurysm repair; IQR, interquartile range; MRI, magnetic resonance imaging; SD, standard deviation; TAA, thoracic aortic aneurysm.

**Table 3:** Contingency tables of true-FISP, HASTE, and MRI (true-FISP and HASTE combined) assessments for endoleaks compared to CTA.

	CTA Positive	CTA Negative	Total
True-FISP			
Positive	19	21	40
Negative	0	5	5
Total	19	26	45
HASTE			
Positive	19	19	38
Negative	0	7	7
Total	19	26	45
MRI			
Positive	19	21	40
Negative	0	5	5
Total	19	26	45

CTA—computed tomography angiography.

**Table 4:** Comparison of MRI characteristics between patients with a positive and negative CTA.

	CTA Positive	CTA Negative	*p*
Age (median and IQR)	72 (67–76)	76 (69–79)	0.490
Thrombus flow in MR + patients (L/min; median, IQR)	0.06 (0.03–0.23)	0.01 (0.01–0.04)	0.007 *
Thrombus flow in all patients (L/min; median, IQR)	0.06 (0.03–0.23)	0.01 (0.01–0.08)	0.016 *
Thrombus/aneurysm ratio (median, IQR)	0.75 (0.66–0.80)	0.70 (0.62–0.78)	0.587
CNR hyperintensity–thrombus true-FISP (median, IQR)	31.67 (22.08–46.57)	39.05 (21.74–50.64)	0.778
CNR hyperintensity–thrombus HASTE (median, IQR)	19.41 (13.88–28.64)	23.93 (16.11–56.04)	0.300

CNR, contrast-to-noise ratio; CT+, CT assessment positive for endoleak; CT−, CT assessment negative for endoleak; * denotes statistical significance.

## Data Availability

Data are available upon reasonable request to the corresponding author.
